# SNP variation landscape of *BMP15* gene from 75 global sheep breeds and their genetic effect on lambing traits

**DOI:** 10.3389/fvets.2025.1612263

**Published:** 2025-07-14

**Authors:** Peiyao Liu, Chunna Cao, Xiangding Wang, Kewei Li, Ebadu Areb, Ran Li, Qingfeng Zhang, Chuanying Pan, Xianyong Lan

**Affiliations:** ^1^Key Laboratory of Animal Genetics, Breeding and Reproduction of Shaanxi Province, College of Animal Science and Technology, Northwest A&F University, Yangling, Shaanxi, China; ^2^Yangling Vocational and Technical College, Yangling, Shaanxi, China; ^3^Tianjin Aoqun Sheep Industry Academy Company, Tianjin, China

**Keywords:** sheep, *BMP15* gene, live lamb number, whole genome sequencing, SNP

## Abstract

**Introduction:**

Bone morphogenetic protein 15 (*BMP15*) is a pivotal regulator of reproductive performance. This study aimed to investigate genetic variations in the sheep *BMP15* gene and their role in lambing traits.

**Methods:**

Whole-genome sequencing was performed on 2,409 individuals from 75 global sheep breeds to identify functional variations in *BMP15*. Variant annotation, protein structure prediction, haplotype analysis, and association studies with lambing traits were conducted.

**Results:**

139 SNPs were detected, including 10 exonic variants (6 missense, 2 synonymous, 2 premature stop codons), 9 of which were novel. The high-frequency missense mutation g.54284258A>G (L251P) showed predicted localized conformational changes. Low-frequency stop-gain mutations (e.g., g.54284551C>T) were rare (<0.005%) in domestic breeds. Haplotype H6 (AAAA) predominated in domestic breeds (48.4%), diverging significantly from the wild ancestral H1 (AAGA), indicating artificial selection. The g.54284258A>G variant was significantly associated with live lamb number at first (*p* < 0.01) and second parity (*p*  < 0.05). AG genotypes had more live lambs than AA (1.29 vs. 1.12; *p* < 0.01). No significant associations were found with total lambing number, lamb survival rate, stillbirth count, or stillbirth rate.

**Discussion:**

*BMP15* exhibits substantial genetic variability shaped by selection. The g.54284258A>G variant significantly influences live lamb numbers and may serve as a genetic marker for improving this trait in sheep breeding programs, though its impact is parity-specific and unrelated to stillbirth or overall survival metrics.

## 1 Introduction

Sheep populations have dispersed across diverse ecology worldwide after domestication, encountering heterogeneous environmental pressures and selective constraints that resulted adaptive diversification through genetic variation (1). This evolutionary process, underpinned by the emergence of advantageous alleles, has shaped the species’ global genetic diversity, highlighting the importance of characterizing genetic variability for both conservation of unique breeds and optimization of breeding programs (2). While advances in reproducible genotyping tools have enabled cross-border collaborations for in-depth study of genetic diversity (3).

Reproductive traits, particularly litter size (total lambs born including stillbirths) and number of live lambs (viable neonates surviving >24 h postpartum), are polygenic in nature, modulated by intricate interactions among environmental factors (e.g., nutrition and management), epigenetic modifications, and multi-gene regulatory networks. Their low heritability (*h*^2^ = 0.1–0.3) renders conventional phenotypic selection strategies ineffective for achieving genetic gains (4). This limitation underscores the urgency to decipher the genetic regulatory networks governing these traits and identify causal functional mutations. It might be prerequisite for developing precision breeding technologies aimed at enhancing ovine reproductive efficiency (including litter size, live lambs and others).

Bone morphogenetic protein 15 (*BMP15*), an X-linked oocyte-specific member of the TGF-β superfamily, has emerged as a pivotal regulator of reproductive performance. Expressed from the early secondary follicular stage through ovulation (5). *BMP15* influences folliculogenesis and embryo survival via conserved signaling pathways. Although mutations such as FecXI and FecXR are associated with prolificacy in select sheep breeds (6). On the other hand, *BMP15* variants show no significant association with litter size in Indian Black Bengal goats (7) and Tibetan cashmere goats (8). These discrepancies suggest species- and breed-specific functional roles for *BMP15*, potentially modulated by genetic background or environmental covariates. Therefore, systematic validation across globally diverse populations is essential.

This study integrated high-throughput sequencing with functional gene annotation to systematically identify genetic variants associated with litter size and number of live lambs, while evaluating their distribution patterns and potential effects across diverse sheep breeds. Through comprehensive analyses, we aim to screen candidate genes and loci significantly associated with lambing traits, providing novel insights into the genetic basis for reproductive performance. These findings contribute to understanding of the genetic regulation of complex traits and lay scientific foundation for molecular breeding strategy to enhance sheep reproductive efficiency.

## 2 Materials and methods

All experimental procedures were performed in accordance with the Faculty of Animal Policy and Welfare Committee of Northwest A&F University (protocol no. NWAFU-314020038) for the use and care of animals in research.

### 2.1 Samples collection

This study utilized whole-genome sequencing data from 2,409 sheep individuals publicly available in the NCBI SRA database^1^ (Supplementary Table S1), with an average sequencing depth of 13.25×. These samples represent 75 global sheep breeds, including 361 individuals from Africa, 339 from America, 1,021 from East Asia, 587 from Europe, 52 from the Middle East, and 49 wild sheep (*Ovis orientalis*) (Supplementary Table S1).

Lambing traits such as litter size, number of live lambs, live lamb rate, stillbirth count, still birth rate, and lamb survival rate was collected from Australian White (AUW) sheep (*n* = 576) in Tianjin, China. Approximately 8 mL blood sample was collected from each ewe into EDTA-coated tubes from jugular vein and stored at −80°C for subsequent DNA extraction. Phenol-chloroform extraction method was used to extract genomic DNA from blood. The concentrations were measured by a Nanodrop 2000 Spectrophotometer to assess DNA purity (A_260_/_280_ ratio) and quality, and were diluted to 20 ng/μL and frozen at −40°C for further experiments. To minimize genetic confounding effect, unrelated individuals were selected based on pedigree records. Additionally, fixed effects such as age and parity were collected.

### 2.2 Read alignment and SNP detection

Raw FASTQ files underwent initial quality filtering using fastp v0.20.0. The processed reads were then aligned to the *Ovis aries* reference genome, ARS-UI_Ramb_v2^2^ using BWA-MEM v0.7.13-r1126, generating binary alignment (BAM) files. Variant calling was performed using GATK v3.6-0-g89b7209, leveraging the “HaplotypeCaller” and “GenotypeGVCFs” modules to identify single nucleotide polymorphisms (SNPs). To ensure data quality, subsequent filtering was conducted with bcftools-1.13, applying stringent thresholds to remove artifacts: QD < 2.0, QUAL < 30.0, SOR > 3.0, FS > 60.0, MQ < 40.0, MQRankSum < −12.5, and ReadPosRankSum < −8.0. Further filtering retained only biallelic SNPs (-m2 -M2 -i) with a minor allele frequency (MAF) ≥ 0.05 and a genotype missing rate (F_MISSING) < 0.2.

### 2.3 Variant annotation

Gene annotation was performed using SnpEff v4.2 (9), categorizing variants into intronic, exonic, intergenic, splice-site, upstream, and downstream regulatory regions. SNPs located in exonic regions were further classified as synonymous or nonsynonymous variants. Finally, VCFtools v0.1.16 (10) was employed to calculate allele frequencies for all SNPs across different sheep breeds.

### 2.4 Linkage disequilibrium analysis

Linkage disequilibrium (LD) analysis was performed for all SNPs within the coding regions of the *BMP15* gene using the LDheatmap R package (11).

### 2.5 Protein structure prediction

The amino acid sequences of the ovine *BMP15* gene (NP_001108239.1) were retrieved from the NCBI Protein database.^3^ Three-dimensional protein structures were predicted using the AlphaFold2 online tool,^4^ with structural modeling performed for both wild-type and missense mutation variants. Finally, PyMOL v7.0.1 (12) was used to visualize and compare the protein structures before and after mutation.

### 2.6 Haplotype analysis

VCF files for the coding regions of the *BMP15* gene were first filtered using bcftools v1.13 (10) with criteria of minor allele frequency (MAF) > 0.1 and genotype missing rate < 0.1. The filtered VCF files were subsequently converted to FASTA format. Multiple sequence alignment was then performed using MAFFT (13). Haplotype identification was streamlined by extracting haplotype data (.rdf files) from the aligned FASTA sequences using DnaSP v6.0 (14). Finally, low-frequency haplotypes (frequency < 5%) were excluded, and haplotype networks were visualized using Network v10.2 (15).

### 2.7 KASP genotyping

KASP (Kompetitive Allele-Specific PCR) primers were designed for the identified missense SNP 54284258 A>G loci. KASP primer set included a common reverse primer and two allele-specific forward primers differentiated by their 3′-terminal nucleotides to target alternate SNP alleles (16). Primers were synthesized by Sangon Biotech (Shanghai, China) Co., Ltd.; sequences are detailed in Table 1.

**Table 1:** KASP primers pairs for *BMP*15-54284258 genotyping.

Locus	Primer type	Sequence (5′ → 3′)
ChrX:54284258 A>G	FAM primer	GAAGGTGACCAAGTTCATGCTGGGTCTTTTTCTGTAAACTCTTTCA
	VIC primer	GAAGGTCGGAGTCAACGGATTGGGTCTTTTTCTGTAAACTCTTTCG
	Common reverse primer	TGACACTCAGAGTGTTCAGAAGACCAAAC

Chr, chromosome; FAM and VIC: Two Fluorescent Reporter Dyes.

The KASP reaction comprises two primary components, Primer Mix and Master Mix. The Primer Mix consists of forward and reverse primers pooled at specified concentrations. The Master Mix contains universal detection primers labeled with distinct fluorophores (e.g., FAM and VIC). PCR amplification for genotyping used a reaction mixture with a total volume of 10 μL with 2.5 μL ddH_2_O, 0.5 μL of primer mix (FAM/VIC/R primers), 2 μL of DNA, and 5 μL of master mix (17). The cycling temperature adjustment with the respective number of cycles was described in Table 2.

**Table 2:** KASP reaction protocol.

Steps	Procedures	Temperature	Time	Cycles
Step 1	Initial denaturation	95°C	10 min	1
Step 2	Touchdown PCR*	95°C	15 s	10
		61 → 55°C (Δ = −0.6/cycle)	45 s	
Step 3	Amplification	95°C	15 s	28–35
		55°C	45 s	
Step 4	Fluorescence plate read	30°C	25 s	1

### 2.8 Data analysis

The association analysis between lambing traits and the g.54284258A>G SNP of *BMP15* gene was analyzed by using the general linear model of SPSS (IBM, 2019). The fixed effects considered were parities and genotype of the ewe. The linear model was Y_jkl_ = μ + A_j_ + G_k_ + e_jkl_, where Y_jkl_ = lambing trait, μ = population mean, A_j_ = effect of parities of ewe (5 levels; from party 1 to parity 5), G_k_ = effect of genotype (3 levels), and e_jkl_ = random error. Statistical significance is defined as *p* < 0.05, and high significance is defined as *p* < 0.01.

## 3 Results

### 3.1 Variant annotation of *BMP15*

**Figure 1:**
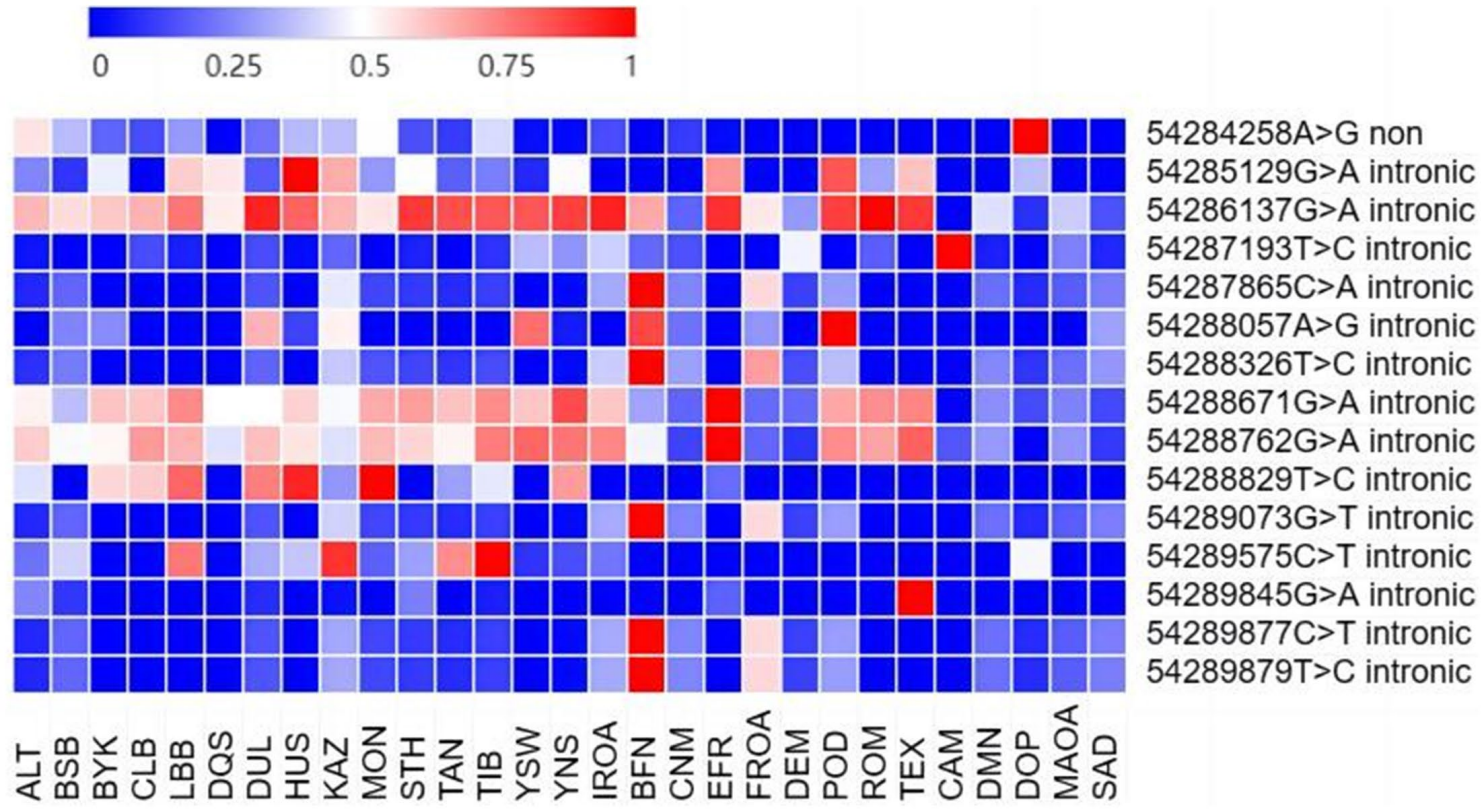
Frequency of SNP variation in *BMP15* gene in different sheep populations. Only those populations with sample size >20 and MAF > 0.01 are displayed in figure; ALT, Altay sheep; BSB, Bashibai sheep; BYK, Bayinbuluke sheep; CLB, Cele Black sheep; LBB, Liangshan black sheep; DQS, Diqing sheep; DUL, Duolang sheep; HUS, Hu sheep; KAZ, Kazakh sheep; MON, Mongolian sheep; STH, Small Tail Han sheep; TAN, Tan sheep; TIB, Tibetan sheep; YSW, Yunnan semi-fine wool sheep; YNS, Yunnan sheep; IROA, Iran Local breed sheep; BFN, Baikal Fine-Fleeced sheep; CNM, Chinese Merino sheep; EFR, East Friesian Dairy sheep; FROA, France local populations sheep; DEM, German merino sheep; POD, Poll Dorset sheep; ROM, Romney sheep; TEX, Texel sheep; CAM, Cameroon; DMN, D’man; DOP, Dorper sheep; MAOA, Morocco local populations; SAD, Sardi sheep.

The *BMP15* gene is located on the sheep X chromosome (positions 54,283,635–54,290,315 bp). Whole-genome sequencing data from 2,409 sheep were analyzed, identifying 139 SNPs within the *BMP15* locus (Supplementary Table S2). Of these, 129 SNPs were localized to intronic regions, while 10 SNPs were exonic (Table 3; Figure 1). Among the exonic variants, 6 were missense mutations, 2 were synonymous mutations, and 2 introduced premature termination codons (PTCs).

**Table 3:** SNP variation information in the coding region of sheep *BMP15* gene.

Variants	Names	Regions	Loci	Amino acid changes	Mutation types
g.54284258A>G	FecXG	exon2	c.T752C	L251P	non
g.54284540C>T	–	exon2	c.G470A	S157N	non
g.54284550C>T	–	exon2	c.G460A	V154I	non
g.54284551C>T	–	exon2	c.G459A	W153X	Stopgain
g.54284552C>T	–	exon2	c.G458A	W153X	Stopgain
g.54284557C>T	–	exon2	c.G453A	E151E	syn
g.54284559C>T	–	exon2	c.G451A	E151K	non
g.54284560C>T	–	exon2	c.G450A	V150V	syn
g.54284562C>T	–	exon2	c.G448A	V150M	non
g.54284567C>T	–	exon2	c.G443A	C148Y	non

syn refers to synonymous mutations, non for missense mutations and stop gain for premature termination. Loci: SNP mutation sites within the gene. Amino acid changes: Corresponding amino acid variations.

Notably, the SNPs g.54286137G>A, g.54288671G>A, and g.54288762G>A exhibited allele frequencies exceeding 0.5 in 75% of the studied populations. In contrast, g.54289877C>T was not detected (frequency = 0) in most breeds but was present at a frequency exceeding 0.1 in the Altay, Valley Tibetan, and Baikal Fine-Fleeced sheep.

Notably, 9 out of 10 exonic SNPs identified in this study are novel, with only g.54284258A>G having been previously reported (Table 3). The potential functional implications of these newly identified variants need further investigation.

### 3.2 Prediction of key mutation impact on protein structure

**Figure 2:**
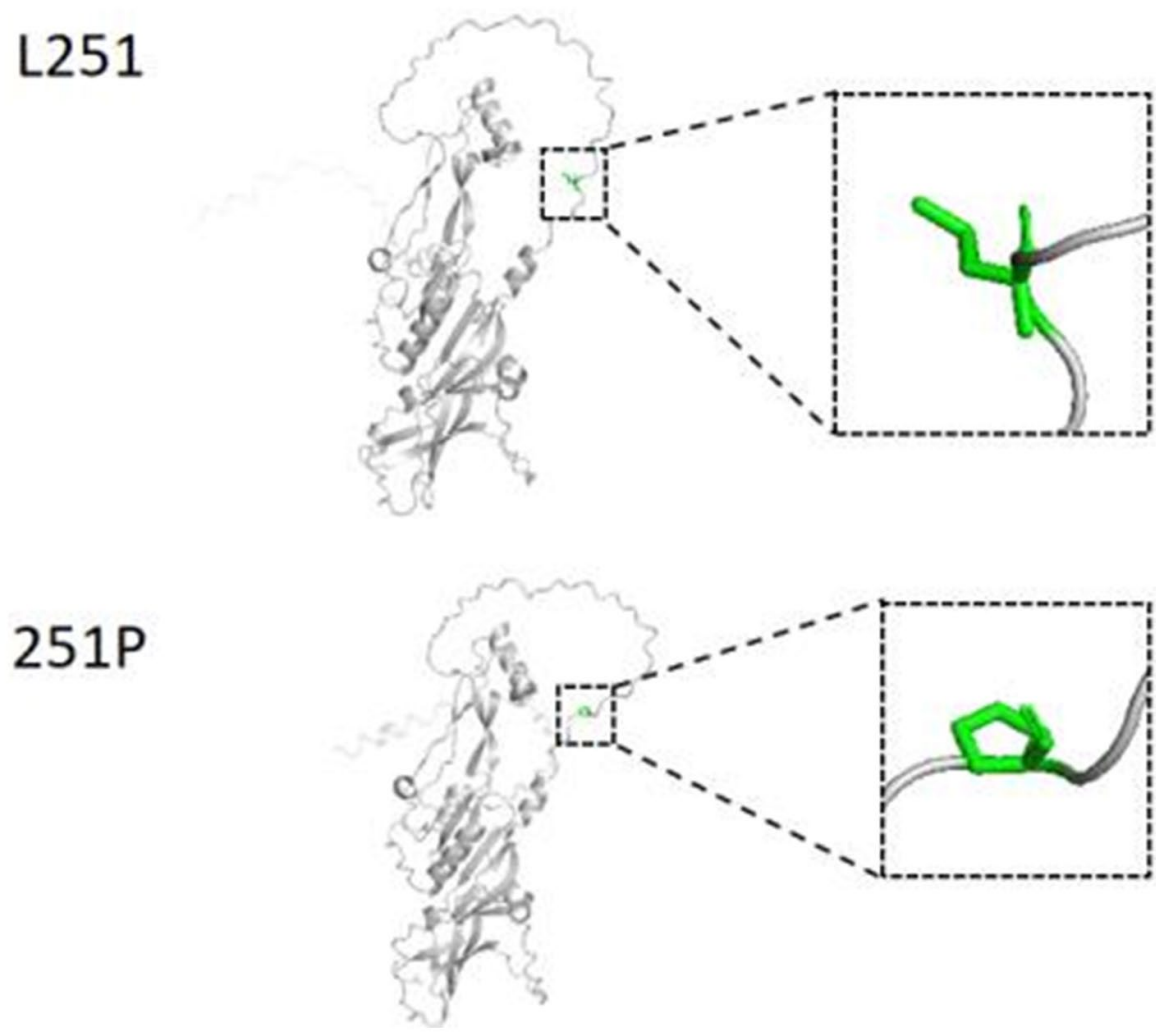
Three-dimensional protein structure changes of L251P before and after mutation. L251, leucine amino acid position at 251 of *BMP15*; 251P, proline amino acid position at 251 after mutation.

A high-frequency missense mutation (MAF > 0.05) was identified within the *BMP15* gene. To evaluate its potential biological impact, the L251P amino acid substitution (leucine to proline at position 251) was modeled using PyMOL v7.0.1, and the resulting protein tertiary structure was compared to the wild-type (Figure 2). Structural predictions revealed detectable alterations in the three-dimensional arrangement of residues near the mutation site, suggesting that this substitution may affect *BMP15* function through interference of local folding.

### 3.3 Haplotype analysis

**Figure 3:**
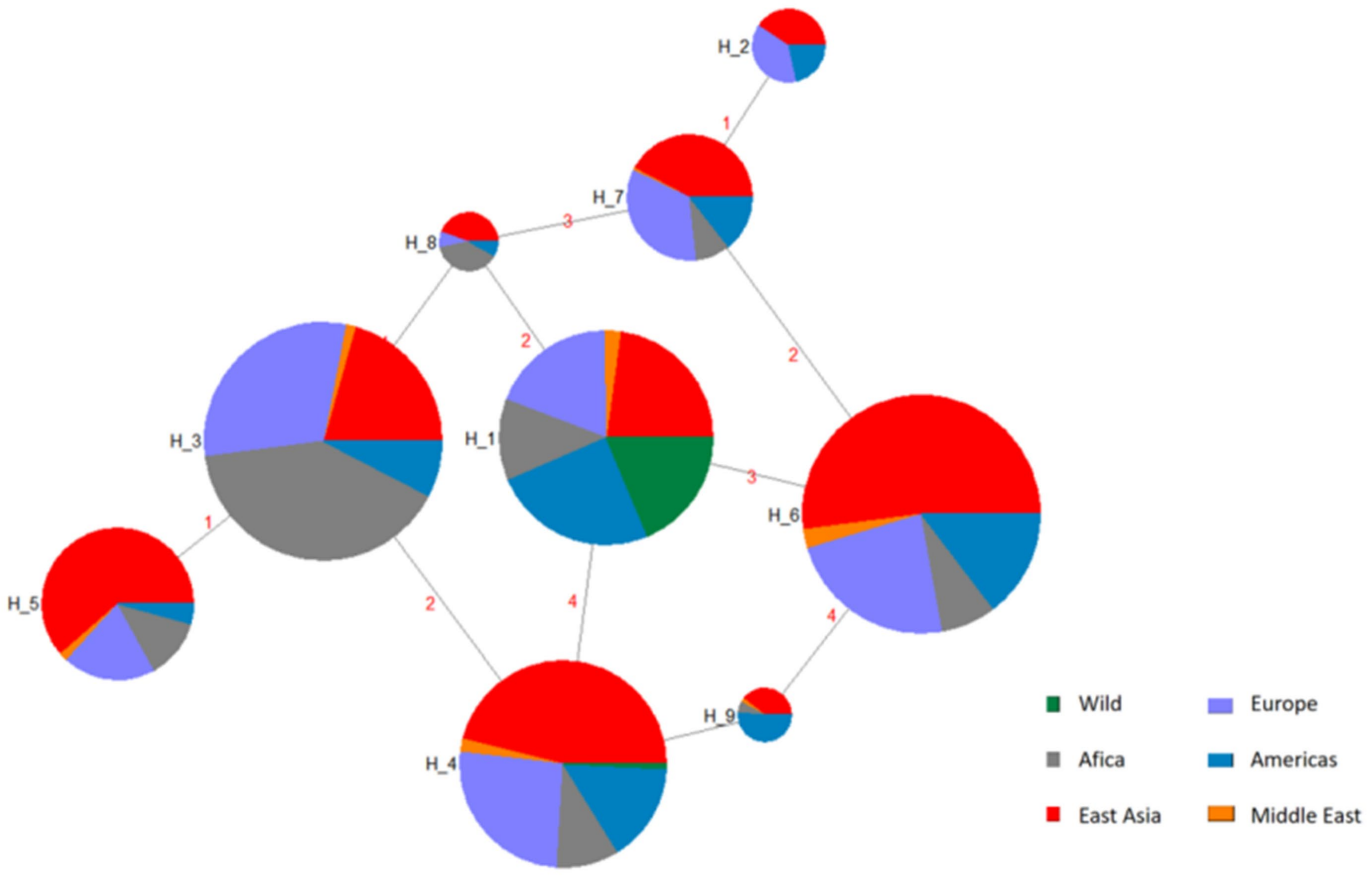
Haplotype networks inferred from SNP mutations in the *BMP15* gene, H1 = AAGA, H2 = GGAA, H3 = AGGG, H4 = AAGG, H5 = GGGG, H6 = AAAA, H7 = AGAA, H8 = AGGA, H9 = AAAG. The network connects the haplotypes with lines which indicate their relationship. On the other hand, size of circles indicates frequency of each haplotype.

Four SNPs in the *BMP15* gene with MAF > 0.05 such as g.54284258A>G, g.54286137G>A, g.54288671G>A, and g.54288762G>A were used to reconstruct haplotypes. Analysis revealed nine haplotypes (H1–H9), with H6 exhibiting the highest global frequency (0.484) across domestic sheep populations (Table 4; Figure 3). The H1 haplotype was exclusively observed in wild sheep populations (e.g., Argali and Mouflon), strongly supporting its designation as the ancestral haplotype, while H2–H9 represent derived haplotypes arising through mutational events during domestication.

**Table 4:** Inferred haplotype frequency of *BMP15* gene.

Haplotype names	Haplotype types	Haplotype frequencies	Sample size
H1	AAGA	0.106	254
H2	GGAA	0.013	31
H3	AGGG	0.195	467
H4	AAGG	0.098	234
H5	GGGG	0.053	127
H6	AAAA	0.484	1,157
H7	AGAA	0.036	86
H8	AGGA	0.008	19
H9	AAAG	0.007	17

### 3.4 Association analysis of *BMP15* g.54284258A>G with lambing traits in Australian white sheep

**Figure 4:**
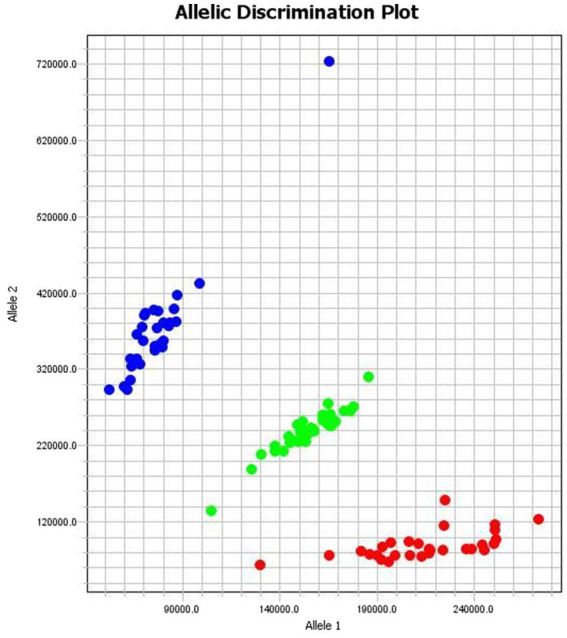
The KASP genotyping results of the g.54284258A>G locus of the sheep *BMP15* gene GG in red, AG in green and AA in blue color. Allele 1 at X-axis and Allele 2 at Y-axis represent fluorescence intensities corresponding to two different alleles for each sample. The clear separation between the three clusters indicates that the genotyping has successfully distinguished between the different genotypes.

Genotyping of the Australian White sheep population at the *BMP15* locus g.54284258A>G was performed using fluorescence signal scanning. The three genotypes (AA, AG, and GG) formed three distinct clusters in the scatter plot (Figure 4), demonstrating high-resolution discrimination and validating the accuracy of the KASP assay.

The *BMP15* g.54284258A>G locus showed a significant association of number of live lambs with first and second parities at (*p* < 0.01) and (*p* < 0.05), respectively (Table 3). For most parities, the G allele (in both AG and GG genotypes) results in a better number of live lambs than the A allele, except in the fourth parity (Supplementary Table S3). Therefore, the *BMP15* g.54284258 A>G variant could be used as a marker for marker-assisted selection to improve the reproductive efficiency of sheep.

## 4 Discussion

This study identified 139 SNPs within the *BMP15* locus in sheep, including 10 exonic variants which comprised 6 missense mutations, 2 synonymous mutations, and 2 premature termination codon (PTC)-introducing mutations. Notably, 9 out of 10 exonic SNPs were novel discoveries, with only g.54284258A>G (FecXG) having been previously reported. The PTC mutations g.54284551C>T (p.W153X) and g.54284552C>T (p.W153X) are predicted to result in complete loss of *BMP15* function, analogous to the fertility-impairing effects of known FecX mutations (e.g., FecX^I^, FecX^L^) (18). However, these novel truncating mutations exhibited extremely low frequencies in domestic sheep (MAF < 0.005), suggesting potential negative impacts on reproductive efficiency and subsequent purging via natural or artificial selection.

The high-frequency missense mutation g.54284258A>G (p.L251P) was predicted to alter local protein conformation. This discrepancy may be due to two potential mechanisms. First, X-linked dosage effects could lead to partial compensation in heterozygous (AG) females compared to homozygous (AA/GG) individuals, a phenomenon mediated by X-chromosome inactivation (19). Second, indirect mechanisms might operate, where the mutation modulates follicular atresia to influence fetal survival rather than directly increasing ovulation rate—a hypothesis supported by its significant association with first-parity live lamb number (*p* < 0.01) (20).

Haplotype analysis of four high-frequency SNPs delineated nine haplotypes (H1–H9), with H6 (AAAA) dominating global domestic sheep populations (48.4% frequency), while wild mouflon sheep retained the ancestral haplotype H1 (AAGA). The prevalence of H6 likely reflected long-term artificial selection for enhanced fecundity: *BMP15* in wild populations balances ovulation rates to avoid maternal energy depletion, whereas domestication favored haplotypes promoting higher prolificacy. Interestingly, H3 (AGGG) showed elevated frequency in East Asian breeds (19.5%), suggesting potential adaptive advantages in local environments, though functional validation is required.

While g.54284258A>G showed no association with total litter size, its significant effect on number of live lambs (1.29 vs. 1.12 lambs for AG vs. AA genotypes in first parity, *p* < 0.01) (Table 5) suggested *BMP15’*s regulatory role in embryonic survival. This dual functionality might originate from two mechanisms. First, *BMP15*-mediated inhibition of granulosa cell apoptosis, which promotes follicular maturation (21). Second, *BMP15* signaling optimization of placental angiogenesis, thereby enhancing fetal nutrient supply and postnatal viability (22). Thus, AG/GG genotypes may improve lamb survival through enhanced placental efficiency rather than direct increases in ovulation. Notably, the enhancing effect of AG/GG genotypes on number live lambs was confined to early parities, with no statistically significant differences observed in subsequent parities. This divergence might be explained by two combined mechanisms. First, maternal life-history trade-offs between reproductive efficiency and survival may lead primiparous individuals to prioritize nutrient allocation to fetal development over self-maintenance, adopting a ‘high-care strategy’ to maximize offspring survival (23). Second, diminished statistical power caused by reduced sample sizes in later parities likely obscured subtle genotypic effect. Therefore, future research should employ large-scale, age-stratified cohort designs with sufficient sample sizes to elucidate the complex interactions among genetic, epigenetic, and environmental factors driving parity-specific phenotypic outcomes.

**Table 5:** Association analysis between the g.54284258A>G locus of the *BMP15* gene and the number of live lambs in Australian white sheep.

Traits	Observed values (Mean ± SE)			*p* values
	AA	AG	GG	
1st Parity	1.12 ^b^ ± 0.04 (*n* = 169)	1.29 ^a^ ± 0.03 (*n* = 269)	1.25 ^ab^±0.05 (*n* = 138)	0.008**
2nd Parity	1.18 ^b^ ± 0.04 (*n* = 143)	1.33 ^a^ ± 0.04 (*n* = 210)	1.33 ^a^ ± 0.06 (*n* = 108)	0.036*
3rd Parity	1.21 ± 0.06 (*n* = 92)	1.37 ± 0.05 (*n* = 144)	1.39 ± 0.06 (*n* = 80)	0.099
4th Parity	1.45 ± 0.08 (*n* = 44)	1.41 ± 0.08 (*n* = 71)	1.30 ± 0.09 (*n* = 50)	0.466
5th Parity	1.29 ± 0.11 (*n* = 28)	1.49 ± 0.11 (*n* = 37)	1.35 ± 0.12 (*n* = 26)	0.419

Data are presented as mean ± SE. Different superscript letters (^a, b^) within a row indicate significant differences between genotypes (*p* < 0.05, Tukey’s post-hoc test). Shared letters (e.g., ^ab^) denote no significant difference from groups with the respective letters. No superscripts indicate no significant differences among groups. **p < 0.01, *p < 0.05.

These findings underscore that functional variants in highly conserved reproductive genes like *BMP15* may exist as rare alleles, necessitating large-scale cross-breed meta-analyses for detection. On the other hand, the current research limitations and due attention for future related study included the sample size should be as large as possible, a lack of functional validations like *in vitro* assays for p.W153X effects on protein secretion, and unable to address epigenetic factors.

## 5 Conclusion

This comprehensive study elucidates the significant genetic variation landscape within the BMP15 gene across 75 global sheep breeds and its pivotal role in lambing performance, particularly live lamb number. Whole-genome sequencing revealed 139 SNPs, including novel functional variants (6 missense, 2 synonymous, and 2 stop-gain).

Haplotype analysis demonstrated a pronounced divergence between domestic sheep and their wild ancestors, with the H6 (AAAA) haplotype dominating global domestic populations (48.4%), suggesting strong artificial selection for enhanced reproductive efficiency during domestication. Critically, the association analysis established the missense mutation g.54284258A>G (L251P) as a significant genetic marker, specifically linked to increased number of live lambs in both first (*p* < 0.01) and second (*p* < 0.05) parities. Ewes carrying the AG genotype produced significantly more live lambs (1.29 vs. 1.12) compared to the AA genotype in the first parity.

While not associated with total litter size, this finding underscores BMP15’s crucial role in influencing postnatal viability, potentially through mechanisms supporting embryonic/placental development. Collectively, the identified genetic variation, ancestral haplotype shift, and the validated association of the L251P variant with key lambing traits highlight BMP15’s considerable potential as a candidate gene for marker-assisted selection to augment reproductive efficiency and live lamb output in sheep breeding programs. Future studies should focus on functional validation of novel variants, expanding association analyses with larger cohorts encompassing diverse breeds and parities, and exploring potential epistatic or epigenetic interactions.

## Data Availability

The original contributions presented in the study are included in the article/Supplementary material, further inquiries can be directed to the corresponding authors.

## Footnotes

^1^ https://www.ncbi.nlm.nih.gov/sra/

^2^ https://www.ncbi.nlm.nih.gov/datasets/genome/GCA_016772045.1/

^3^ https://www.ncbi.nlm.nih.gov/protein/NP_001108239.1/

^4^ https://colab.research.google.com/github/sokrypton/ColabFold/blob/main/AlphaFold2.ipynb
